# Locus-Specific and Stable DNA Demethylation at the *H19*/*IGF2* ICR1 by Epigenome Editing Using a dCas9-SunTag System and the Catalytic Domain of TET1

**DOI:** 10.3390/genes15010080

**Published:** 2024-01-08

**Authors:** Claudia Albrecht, Nivethika Rajaram, Julian Broche, Pavel Bashtrykov, Albert Jeltsch

**Affiliations:** Institute of Biochemistry and Technical Biochemistry, University of Stuttgart, Allmandring 31, 70569 Stuttgart, Germany; claudia.albrecht@ibtb.uni-stuttgart.de (C.A.);

**Keywords:** epigenome editing, imprinting, DNA demethylation, dCas9, SunTag, TET1, ICR

## Abstract

DNA methylation is critically involved in the regulation of chromatin states and cell-type-specific gene expression. The exclusive expression of imprinted genes from either the maternal or the paternal allele is regulated by allele-specific DNA methylation at imprinting control regions (ICRs). Aberrant DNA hyper- or hypomethylation at the ICR1 of the *H19/IGF2* imprinting locus is characteristic for the imprinting disorders Beckwith–Wiedemann syndrome (BWS) and Silver–Russell syndrome (SRS), respectively. In this paper, we performed epigenome editing to induce targeted DNA demethylation at ICR1 in HEK293 cells using dCas9-SunTag and the catalytic domain of TET1. 5-methylcytosine (5mC) levels at the target locus were reduced up to 90% and, 27 days after transient transfection, >60% demethylation was still observed. Consistent with the stable demethylation of CTCF-binding sites within the ICR1, the occupancy of the DNA methylation-sensitive insulator CTCF protein increased by >2-fold throughout the 27 days. Additionally, the *H19* expression was increased by 2-fold stably, while *IGF2* was repressed though only transiently. Our data illustrate the ability of epigenome editing to implement long-term changes in DNA methylation at imprinting control regions after a single transient treatment, potentially paving the way for therapeutic epigenome editing approaches in the treatment of imprinting disorders.

## 1. Introduction

DNA methylation and hydroxymethylation, as well as histone post-translational modifications and non-coding RNAs, constitute the epigenome that regulates chromatin states and gene expression in differentiated cell types of multicellular organisms [1]. In eukaryotes, DNA methylation occurs at the C5 position of cytosine (5mC), mostly in the context of CG sequences, and plays an important role in gene expression, gene silencing, and genomic imprinting [2,3]. Unmethylated CpG islands are present in most human promoters of actively transcribed genes, whereas DNA methylation at the promoters can lead to the transcriptional silencing of the associated genes and at repetitive elements [4,5]. DNA methylation is catalyzed by DNA methyltransferases that use S-adenosyl-L-methionine as a methyl group donor [6]. The active removal of DNA methylation is initiated by the ten-eleven translocation (TET) protein family of dioxygenases, catalyzing the iterative oxidation of 5-methylcytosine (5mC) to 5-hydroxymethylcytosine (5hmC), 5-formylcytosine (5fC), and 5-carboxylcytosine (5caC) [7,8,9], which is followed by the removal of the oxidized bases 5fC and 5caC catalyzed by Thymine-DNA glycosylase [10].

During normal development, DNA methylation patterns are established in early embryogenesis and largely maintained afterwards, with small tissue-specific adaptations [2,3]. However, in recent years, epigenome editing technologies have been developed, allowing the change in cellular DNA methylation levels at specific target loci. For this, DNA methylation-modifying effector domains are recruited to the desired target region through a DNA-binding domain (DBD). The most flexible and commonly used DBD is the nuclease-deactivated CRISPR-associated protein 9 (dCas9), which can bind to genomic targets via a locus-specific single-guide RNA (sgRNA) sequence [11]. Instead of fusing a single effector domain to the DBD, it was shown that multiple single-chain variable fragment (scFv)-fused effectors can be recruited to the same genomic locus by using dCas9 fused to a SunTag [12]. This system was highly efficient and showed a reduced off-target activity in some applications compared to the direct fusion of the DNA methyltransferase DNMT3A effector domain to dCas9 for targeted DNA methylation [13,14]. With a modified linker length between the peptide repeats of the original SunTag, this system achieved very strong DNA demethylation efficiencies using TET1CD as the effector domain [15].

**Figure 1:**
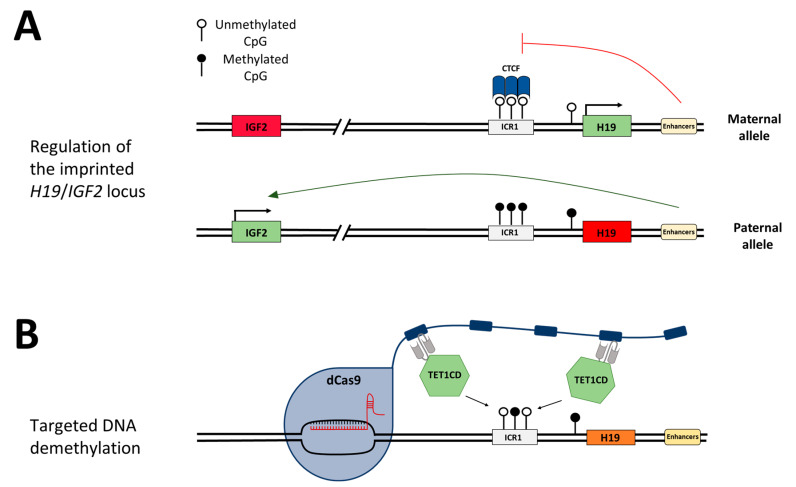
Schematic depiction of the *H19*/*IGF2* imprinting region and the concept of targeted DNA demethylation. (**A**) Schematic regulation of the *H19*/*IGF2* imprinting locus. On the maternal allele, the ICR1 is unmethylated, and the DNA methylation-sensitive CTCF protein can bind and hinder the enhancers from activating *IGF2* expression (red line). *H19* is expressed. On the paternal allele, DNA methylation at the CTCF target regions within the ICR1 hinders CTCF from binding, which provides the enhancers access to promote *IGF2* expression (green arrow). *H19* is silenced by DNA methylation. (**B**) Epigenome editing at the desired genomic locus is mediated by the binding of the sgRNA/dCas9 complex, which carries a SunTag. One antibody-fused effector domain TET1CD can bind to each peptide repeat of the SunTag and mediate DNA demethylation at the target region.

Genomic imprinting is found in about 150 mammalian genes that are frequently involved in growth control and development [16,17]. Imprinted genes are solely expressed from either the maternal or the paternal allele. They often occur in clusters, spanning up to several thousand kilobases [16,18]. The differential expression is maintained by allele-specific DNA methylation at imprinting control regions (ICRs) which is established in germ cells and maintained after fertilization throughout life. Additionally, the clusters usually contain at least one non-coding RNA typically expressed from the maternal allele [16,18]. Errors in the placement or removal of DNA methylation at ICRs can lead to diseases such as cancer, imprinting disorders, and autoimmune, metabolic, or neurological diseases [19,20]. A major cause for the imprinting disorder Silver–Russell syndrome (SRS) is DNA hypomethylation at the imprinting control region 1 (ICR1) on chromosome 11p15, controlling the expression of the insulin-like growth factor 2 gene (*IGF2*) and the H19 non-coding RNA [21,22,23]. SRS is a sporadic and genetically heterogeneous syndrome characterized by intrauterine growth retardation, underweight, a short statue, as well as body asymmetries and a triangular face shape [24]. In healthy individuals, the ICR1 is unmethylated on the maternal allele and *H19* is exclusively expressed from this allele (Figure 1A,B). *IGF2* is silenced through the binding of CTCF (CCCTC-binding factor) to the unmethylated ICR1, which acts as an insulator and hinders downstream enhancers from activating *IGF2* expression. On the paternal allele, *IGF2* is expressed and *H19* is silenced, mediated by DNA methylation at ICR1. The loss of the DNA methylation imprinting mark at the ICR1 in SRS leads to the biallelic repression of *IGF2* and biallelic expression of *H19* [25]. IGF2 has growth-promoting effects; hence, the loss of its expression explains the clinical features of SRS. The imprinting disorder Beckwith–Wiedemann syndrome (BWS) is, contrary to SRS, associated with increased birth weight and somatic overgrowth. Additionally, BWS leads to a predisposition for embryonal malignancies [26]. In 5–10% of diagnosed BWS cases, DNA hypermethylation is found at the ICR1, which causes an opposite gene expression to that observed in SRS patients, namely, the downregulation of *H19* and biallelic expression of *IGF2* [27].

In this study, we aim to demethylate the ICR1 in HEK293 cells to mimic the disease state of SRS and investigate the arising concepts for the treatment of BWS by epigenome editing. For this, we apply an epigenome editing system, in which the target locus is bound by the sgRNA/dCas9 complex fused to the SunTag [12], which in turn recruits the scFv-fused catalytic domain of TET1 (TET1CD) to finally trigger DNA demethylation. We use a two-plasmid system, one containing dCas9 fused to the SunTag with five repeats of the GCN4 peptide, separated by 22 aa long linkers, and scFv-fused TET1CD. The second plasmid encodes five sgRNAs targeting the ICR1 (Supplementary Figure S1). The possibility to demethylate the *H19/IGF2* ICR1 was previously shown in two related epigenome editing studies. In 2016, Morita et al. applied a similar epigenome editing system in embryonic stem cells (ESCs) and observed an almost complete demethylation of four CTCF target sites in ICR1 after treatment, resulting in a significant increase in *H19* expression [15]. In 2020, Horii et al. demonstrated an effective reprogramming of the *H19*/*IGF2* ICR1 in a mouse disease model by applying the epigenome editing system to demethylate ICR1, leading to an upregulation of *H19* and downregulation of *IGF2* expression [25]. In addition, they also applied their system to HEK293 cells. By using six sgRNAs targeting CTCF target -sites (CTSs) and four sgRNAs targeting the *H19* promoter region, they showed significant demethylation at all sgRNA targets five days after transfection [25]. Again, the expression of *H19* was upregulated and *IGF2* expression was downregulated. Furthermore, at day 9 after transfection, they showed increased CTCF occupancy at two targeted CTCF binding sites, as well as in one targeted *H19* promoter region. In parallel, our lab performed similar studies in HEK293 cells. In this paper, we were able to confirm the previously shown reprogramming of the *H19*/*IGF2* imprinting locus. In addition, we document strong demethylation of the ICR1 for up to one month in proliferating HEK293 cells. By applying amplicon-based oxidative bisulfite-sequencing, we demonstrate the appearance of 5hmC at the target regions as a primary editing product generated by the catalytic activity of TET1CD. The CTCF occupancy at all targeted CTSs was permanently increased. We also show an increase in *H19* expression up to day 27 after transfection. Likewise, the downregulation of *IGF2* expression was observed three days after transfection; however, this reduction was not maintained until day 27.

## 2. Materials and Methods

### 2.1. Cloning of the EpiEditor Constructs

The individual sgRNA sequences (Supplementary Table S1) were firstly cloned into the single sgRNA expression vector (Addgene plasmid #210212), and then all five sgRNA sequences together with individual U6 promoters and sgRNA scaffolds were cloned into the multi-sgRNA vector (pMulti-sgRNA-LacZ-DsRed vector, a gift from Yujie Sun, Addgene plasmid #99914), as previously described [28]. For the dCas9-5×SunTag-scFV-TET1CD vector, the dCas9-5×SunTag part was taken from the Addgene plasmid #82560, into which scFv-IRES-GFP was cloned to create the dCas9-5×SunTag-scFv-empty vector. The TET1CD sequence was amplified from the Addgene plasmid #82561 by PCR and inserted into the dCas9-5×SunTag-scFv-empty vector to create the final dCas9-5×SunTag-scFv-TET1CD vector (Supplementary Figure S1).

### 2.2. Cell Culture

Human Embryonic Kidney cells 293 (HEK293) were cultured in Dulbecco’s modified Eagle’s medium (DMEM) supplemented with 10% Fetal Bovine Serum (FBS) (Sigma-Aldrich, Taufkirchen, Germany), 4 mM L-glutamine (Sigma-Aldrich, Taufkirchen, Germany), and 10 mL/L penicillin/streptomycin (Sigma-Aldrich, Taufkirchen, Germany). The cells were incubated at 37 °C, 95% relative humidity, and 5% CO_2_. The cell confluency was maintained under 90% by splitting the cells every 2 or 3 days.

For transfection experiments, 1.4 million cells were seeded into 100 mm cell culture dishes. On the following day, 9000 ng of dCas9-5×SunTag-scFv-TET1CD or of the control plasmid with no effector domain (dCas9-5×SunTag-scFv-empty), 500 ng of sgRNA plasmid (Supplementary Figure S1), and 27 μL of FuGENE HD transfection reagent (Promega, Walldorf, Germany) were mixed in 840 μL serum-free DMEM. After incubating the mixture for 15 min at RT, it was homogenously distributed across the dish. On the following day, the growth medium was replaced with a freshly supplemented DMEM. Three days after transfection, the cells were trypsinized and filtered through a 30 μm pre-separation filter (Miltenyi Biotec, Bergisch Gladbach, Germany). Living, single cells showing both GFP and DsRed fluorescence signals were sorted using the Cell Sorter SF800S (Sony Biotechnology, San Jose, CA, USA). Approximately 0.5–1 × 10^6^ sorted HEK293 cells were used for immediate downstream analysis or cell pellets were snap-frozen with liquid nitrogen and stored at −80 °C. The remaining cells were re-seeded into 6-well plates to harvest at later time points. The harvested cells were subjected to centrifugation for 5 min with 300× *g*, the pellets were washed once with PBS and the cell pellets were snap-frozen with liquid nitrogen and stored at −80 °C.

### 2.3. MBD2-Pulldown-qPCR

Basically, MBD2-pulldown was conducted as described in a previous publication from our laboratory with slight modifications [29]. In detail, genomic DNA (gDNA) was isolated from cell pellets of 0.5–1 million cells using the QIAmp DNA Mini Kit (QIAGEN GmbH, Hilden, Germany), according to the manufacturer’s instructions. The isolated gDNA (approx. 3–10 µg in a 200 µL volume) was sheared using the EpiShear^TM^ probe sonicator (Active Motif, Waterloo, Belgium) (2 mm tip, 40% amplitude, 20 × 20 s pulse, and 30 s pause between each pulse). Subsequently, the sheared gDNA was purified using the NucleoSpin^®^ Gel and PCR Clean-up kit (Macherey-Nagel, Düren, Germany) and eluted in 50 μL ddH_2_O pre-warmed to 70 °C. Per sample, 1 μg of sonicated gDNA and 222 nM of GST-MBD2 protein were mixed in cold binding buffer (50 mM Tris-HCl pH 8.0, 150 mM NaCl, 1 mM EDTA, 0.5% IGEPAL^®^ CA-630, and 2 mM DTT) in a final volume of 250 μL, followed by overnight rotation at 4 °C. On the next day, 50 μL Glutathione Agarose 4B beads (Macherey-Nagel, Düren, Germany) were washed four times with 200 μL cold binding buffer (1 min, 2000× *g*); the supernatant was removed and 50 µL beads were added to each GST-MBD2/gDNA sample. The beads/GST-MBD2/gDNA mixtures were incubated on a rotating wheel at 4 °C for 2 h. After the incubation, the supernatant was removed by centrifugation for 2 min at 2000× *g*. The samples were washed three times with 200 μL cold wash buffer (50 mM Tris-HCl pH 8.0, 500 mM NaCl, 1 mM EDTA, 0.5% IGEPAL^®^ CA-630, and 2 mM DTT) by rotation for 5 min at 4 °C, followed by centrifugation for 2 min at 2000× *g*. The supernatant was discarded, 150 μL of elution buffer (10 mM Tris-HCl pH 8.0, 2000 mM NaCl, and 1 mM EDTA) was added, and the samples were rotated at RT for 15 min. The samples were centrifuged for 2 min at 2000× *g* and the supernatant was stored. The elution step was repeated with 100 μL elution buffer and the supernatant was pooled with the first elution fraction. Using the ChIP DNA Purification Kit (Active Motif, Waterloo, Belgium), the DNA was purified. Elution was performed with 100 μL of the elution buffer provided in the ChIP DNA Purification Kit (Active Motif, Waterloo, Belgium).

The DNA was quantified relative to the input DNA (sonicated gDNA) by qPCR using the CFX96 Real-Time PCR detection system (Bio-Rad Laboratories, Inc., Feldkirchen, Germany). A 1:5 dilution series of input gDNA was prepared for each sample. For each qPCR reaction, 7.5 μL 2× ORA™ See qPCR Probe Mix (highQu, Kraichtal, Germany), 0.4 μL forward primer (10 µM), 0.4 μL reverse primer (10 µM), and 5.7 μL ddH_2_O were used, to which 1 µL of template DNA was added. The qPCR primers are listed in Supplementary Table S1. A non-template control (NTC) was included for every primer pair and each sample was pipetted in technical triplicates. The qPCR program was as follows: 95 °C for 3 min, 40 cycles of 95 °C for 3 s, 57 °C for 20 s, and 72 °C for 4 s, followed by a 65–95 °C ramp (0.5 °C increase per 5 s) to generate a melting curve. The qPCR signals were normalized to input and subsequently to the *SLC6A3* locus, which is fully methylated in HEK293 cells [29].

### 2.4. CTCF-ChIP-qPCR

Crosslinked chromatin immunoprecipitation (ChIP) followed by qPCR was used to quantify the binding of CTCF at the *H19*/*IGF2* ICR. Four million cells were harvested and pelleted by 300× *g* for 5 min. The cell pellets were washed twice in PBS and crosslinked by incubating in a solution of 1% formaldehyde in PBS for 10 min, under constant rotation. Afterwards, the reaction was quenched by adding glycine to a final concentration of 125 mM and rotating at RT for 10 min. The crosslinked cells were pelleted and washed with ice-cold PBS. The cell pellets were snap-frozen in liquid nitrogen and stored at −80 °C.

CTCF-ChIP was performed using the Magna ChIP™ HiSens kit (Catalog No. 17-10460, Merck KGaA, Darmstadt, Germany), according to the manufacturer’s protocol. Isolated crosslinked chromatin from 4 million cells was sheared using the EpiShear^TM^ probe sonicator (Active Motif, Waterloo, Belgium) with the 2 mm tip (40% amplitude, 12 × 20 s pulse, and 30 s pause between each pulse). For each ChIP reaction, 50 or 100 μL of crosslinked and sonicated chromatin and 3 μL of αCTCF antibody (CTCF rabbit AB, Cell Signaling Technology Europe, Frankfurt am Main, Germany, catalog #2899S, Lot: 2) were used. As a control, a rabbit IgG of the same isotype (Normal Rabbit IgG Control, catalog #AB-105-C, 1 μg/μL, R&D Systems, Inc./Bio-Techne GmbH, Wiesbaden-Nordenstadt, Germany) was included. The precipitated DNA was quantified relative to the input DNA by qPCR as described for MBD2-pulldown-qPCR. Subsequently, the qPCR signals were normalized to the average of two control CTCF target sites.

### 2.5. RT-qPCR

Frozen pellets of 0.5–1 million transfected and sorted HEK293 cells were thawed on ice and the RNA was isolated using the RNeasy^®^ Plus Mini Kit (QIAGEN GmbH, Hilden, Germany), according to the manufacturer’s instructions. To synthesize the complementary DNA (cDNA), 500 ng of purified RNA was used with the High-Capacity cDNA Reverse Transcription Kit (Applied Biosystems/Life Technologies GmbH, Darmstadt, Germany). Instead of 10× RT Random Primers, the Oligo d(T)_18_ mRNA Primer (5 A_260_ units, NEB) was used. For each sample, a reaction with reverse transcriptase (plus-RT) and a control reaction without reverse transcriptase (non-RT) were carried out. For the subsequent qPCR, each sample was diluted 1:3 with ddH_2_O. For each RT-qPCR reaction, 7.5 μL 2× ORA™ See qPCR Probe Mix (highQu, Kraichtal, Germany), 0.4 μL forward primer (10 µM), 0.4 μL reverse primer (10 µM), and 4.7 μL ddH_2_O were mixed, followed by the addition of 2 µL cDNA. The RT-qPCR primers are listed in Supplementary Table S1. All samples (plus-RT, non-RT, and non-template controls) were pipetted in triplicate. Plus-RT cDNA samples were pooled and a 1:5 input dilution series was prepared to determine the PCR efficiency. The qPCR was run as follows: 95 °C for 3 min, 40 cycles of 95 °C for 15 s, followed by 57 °C (*SDHA*) or 62 °C (*H19* and *IGF2*) for 30 s, 95 °C for 10 s, and lastly a 65–95 °C ramp (0.5 °C steps every 5 s) to generate the melting curve. The transcript levels of *H19* and *IGF2* were analyzed by normalizing the Cq values to the reference gene Succinate Dehydrogenase Complex Flavoprotein Subunit A (*SDHA*) using the −ΔΔCq calculation as described by Pfaffl (2001) [30].

### 2.6. Bisulfite Sequencing and Oxidative Bisulfite Sequencing

The gDNA of transfected HEK293 cells sorted by FACS was isolated using the QIAmp DNA Mini Kit (QIAGEN GmbH, Hilden, Germany), according to the manufacturer’s instructions. A total of 500 ng gDNA was fragmented enzymatically by overnight digestion using 40 U EcoRV-HF (a non-cutter in the genomic regions desired for amplification) (New England Biolabs GmbH, Frankfurt am Main, Germany) in CutSmart buffer in a total volume of 20 µL. On the next day, bisulfite conversion was conducted using the EZ DNA Methylation-Lightning™ Kit (Zymo Research Europe GmbH, Freiburg, Germany), according to the manufacturer’s protocol. Oxidative bisulfite conversion was performed using the TrueMethyl^®^ oxBS Module (Part No. 0414, Tecan Group Ltd., Männedorf, Switzerland.), according to the manufacturer’s instructions. Bisulfite-converted DNA was eluted in 12 µL ddH_2_O.

The amplicons of interest were amplified in a first PCR1 with locus-specific primers (Supplementary Table S1), which also contained barcodes and adapters complementary to PCR2 primers. For each PCR1 reaction, 1 µL of BS-converted DNA template was mixed with 1 µL forward primer (10 µM), 1 µL reverse primer (10 µM), 14.4 µL ddH_2_O, 2 µL 10× PCR buffer (QIAGEN GmbH, Hilden, Germany), 0.4 µL dNTPs mix (40 mM), and 0.2 µL of HotStarTaq-Polymerase (5 U/µL, QIAGEN GmbH, Hilden, Germany). The PCR thermocycling program was 95 °C for 15 min, 35 cycles of 94 °C for 30 s, Tm for 30 s, and 72 °C for 1 min, followed by 72 °C for 10 min (Tm values are specified in Supplementary Table S2). The amplicons were verified by loading 5 µL of the PCR1 reaction on a 1% agarose gel. The PCR1 product was used as a template for PCR2, in which Illumina TruSeq sequencing indices were added to the amplicons. A total of 1 µL PCR1 product (for most genes, the PCR1 product was diluted with ddH_2_O before proceeding with PCR2) was mixed with 0.8 µL forward primer (10 µM), 0.8 µL reverse primer (10 µM), 12.8 µL ddH_2_O, 4 µL 5× Q5 reaction buffer (New England Biolabs GmbH, Frankfurt am Main, Germany), 0.4 µL dNTPs mix (40 mM), and 0.2 µL of Q5 High-Fidelity DNA Polymerase (New England Biolabs GmbH, Frankfurt am Main, Germany). The PCR thermocycling program was 98 °C for 30 s, 15 cycles of 98 °C for 10 s and 72 °C for 40 s, and lastly 72 °C for 2 min. The PCR2 products were verified by loading 5 µL on a 1% agarose gel. For the final library pooling, the PCR2 amplicons were loaded onto a 0.8% agarose gel run in TAE buffer (1 mM EDTA disodium salt, 40 mM Tris, and 20 mM acetic acid). The bands were excised from the gel and purified using the NucleoSpin^®^ Gel and PCR Clean-up kit (Macherey-Nagel, Düren, Germany), according to the manufacturer’s protocol. Sample concentrations were measured using the NanoDrop 1000 (Thermo Fisher Scientific/Life Technologies GmbH, Darmstadt, Germany) and equimolar amounts of samples were pooled. Paired-end Illumina sequencing with a 250 bp read length was performed by the Novogene (UK) Company Limited, Cambridge, United Kingdom.

### 2.7. NGS Data Processing and Data Analysis

NGS data in a FASTQ format were analyzed as described [28] on the Galaxy platform (https://usegalaxy.org/) [31], where all the following tools are available. In brief, Illumina adapter sequences were removed using Trim Galore! (Galaxy Version 0.4.3.1). Afterwards, two paired-end reads were merged using Pear (Galaxy Version 0.9.6.1) and reads with low quality were removed using Filter FASTQ (Galaxy Version 1.1.1). The de-multiplexing of individual samples tagged with combinations of barcodes and Illumina indices was conducted by converting the FASTQ files using FASTQ to Tabular (Galaxy Version 1.1.5), followed by the selection of lines with the tool Select (Galaxy Version 1.0.1) and the re-conversion of the files to a FASTQ format with Tabular to FASTQ (Galaxy Version 1.1.1). For the alignment of reads to a reference sequence, bwameth (Galaxy Version 0.2.0.4) was used and the DNA methylation at each CpG site was analyzed by applying the tool MethylDackel (Galaxy Version 0.3.0.1). The output files were processed using Microsoft Excel (Professional Plus 2019). DNA methylation at non-CpG sites is known to be much lower in human cell lines and it was not investigated in this paper [32].

## 3. Results

### 3.1. Targeted Demethylation of the H19/IGF2 ICR1 Using dCas9-SunTag and scFv-TET1CD

The targeted alteration of DNA methylation at specific genomic loci can be achieved by applying the dCas9-SunTag system, which recruits multiple copies of the effector module fused to an antibody scFv fragment to the target site. For targeted DNA demethylation, the catalytic domain of the methylcytosine dioxygenase TET1 (TET1CD) can be used. In this study, we used a dCas9-SunTag fusion construct containing five copies of the GCN4 peptide separated by 22 amino acid (aa)-long linkers (Figure 1B). Up to five scFv-fused effector domains could be recruited to one dCas9-SunTag protein, which is directed to the desired target sequence by a sgRNA. We employed the dCas9-SunTag system in HEK293 cells for the locus-specific DNA demethylation of the ICR1 located between the *H19* and *IGF2* genes. At this ICR, DNA methylation is only present on the paternal allele in human cells. After removing the DNA methylation on the paternal allele, we expected to observe downstream effects similar to those seen in SRS patients also carrying a hypomethylated *H19*/*IGF2* ICR, viz., the binding of the insulator protein CTCF to unmethylated CTCF target sites (CTSs) on both alleles, which leads to the biallelic expression of *H19* and the downregulation of *IGF2*.

**Figure 2:**
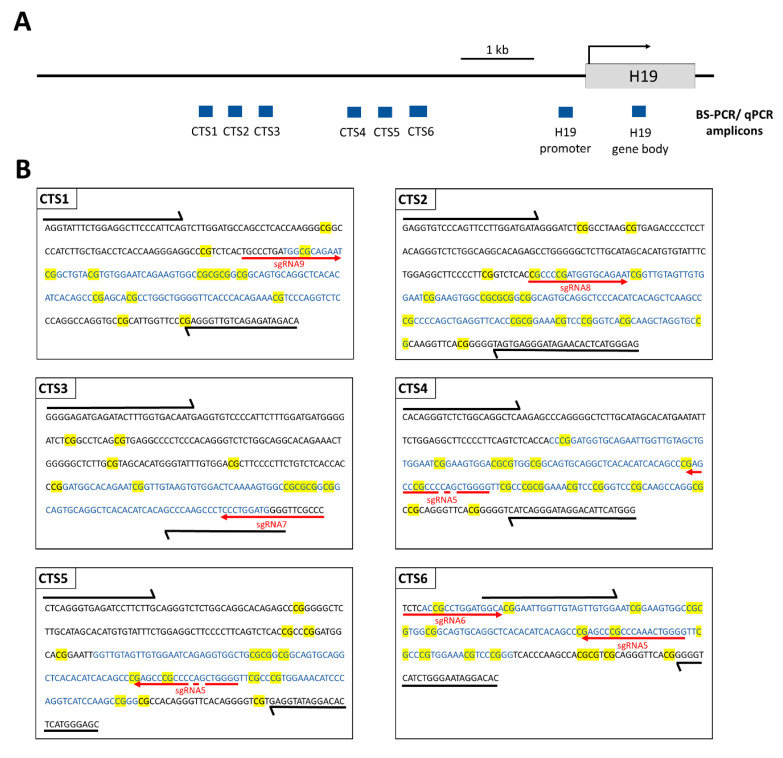
Scheme illustrating the ICR1 and the *H19* gene on chromosome 11p15.5 (chr11: 1,996,336–2,003,171, GRCh38/hg38). (**A**) PCR amplicons used for BS-seq and qPCR (for MBD2-Pulldown and CTCF-ChIP) within the CTCF target sites (CTS) are depicted in blue rectangles. (**B**) The sequences of the PCR amplicons are shown for CTS1-6. The sequences of the HEK293 cell line were obtained from http://hek293genome.org/v2/. Black arrows show the BS-PCR primer binding sites. The qPCR amplicons are represented by blue letters. CpG sites are highlighted in yellow. The respective sgRNA binding sites are shown by red arrows. The primer sequences and genomic positions of the BS-seq amplicons are provided in Supplementary Table S2.

To initiate epigenome reprogramming, the HEK293 cells were co-transfected with two plasmids (Supplementary Figure S1). The first plasmid contained the dCas9-SunTag system, as well as the scFv-fused TET1CD, followed by the *GFP* gene, leading to GFP expression being mediated by an IRES. The second plasmid contained five sgRNAs (sgRNA5–sgRNA9) that target four CTSs (CTS1, CTS2, CTS3, and CTS6) within the ICR1 region, as well as a *DsRed* gene expressed by a separate promoter. The binding sites of the sgRNAs are shown in Figure 2. CTS4 and CTS5 do not contain a perfect binding site, but they are presumably also targeted, since both regions can be bound by sgRNA5 with two mismatches. Three days after transfection, GFP and DsRed double-positive cells were sorted by FACS. On the same day, a part of the cells was harvested for gDNA isolation and the remaining cells were cultivated and harvested on days 6, 9, 15, 21, and 27 after transfection.

**Figure 3:**
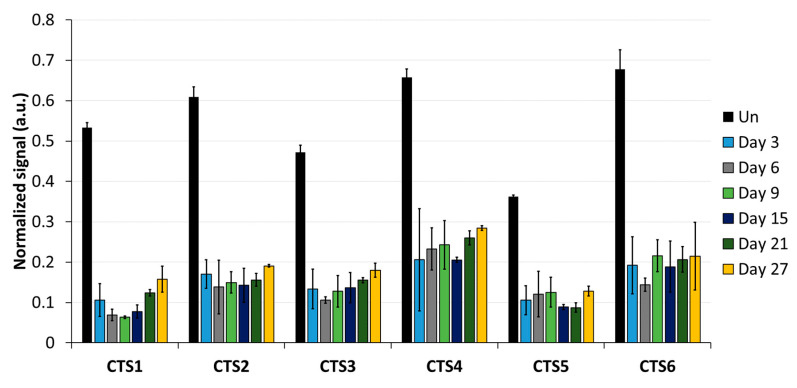
DNA methylation analysis at the CTCF target site (CTS) regions analyzed by MBD2-pulldown. MBD2-pulldown qPCR signals were normalized to the input DNA and to the signal obtained at the fully methylated promoter of *SLC6A3*. Data are shown for CTS1-6 in untreated (Un) cells, as well as for different time points (day 3, day 6, day 9, day 15, day 21, and day 27) after transfection with the epigenome editing system. Data are represented as the mean of three independent biological replicates ± standard deviation (SD) for Un and days 3, 6, and 9. For days 15, 21, and 27, data are shown as the mean of two independent biological replicates ± SD. a.u. = arbitrary unit.

As a first step, the DNA methylation analysis was conducted by MBD2-pulldown coupled with qPCR (Figure 3). The qPCR signals were normalized to the signal observed at the promoter region of *SLC6A3*, which is fully methylated in HEK293 cells [29]. The average methylation levels obtained from normalized MBD2-qPCR signals in untreated cells at CTS1-6 was 55%. This indicates that the pulldown at CTS1-6 is approximately half as intensive as that observed at the *SLC6A3* locus, which agrees with the presumption that half of the alleles are methylated at the imprinted locus. After DNA methylation editing using TET1CD, a drastic loss of MBD2-pulldown at all CTSs was observed, which is indicative of efficient DNA demethylation. Three days after transfection, the strongest effect was seen at CTS1, with only 10.6% of the pulldown signal remaining, which corresponds to a 5-fold reduction compared to what was observed in the untreated cells. On average, the pulldown signal at CTS1-6 was 15.2%, corresponding to 3.6-fold lower than that of the untreated cells, indicating a strong DNA demethylation. Most importantly, the DNA demethylation remained stable up to 27 days after transfection. At this time point, the average MBD2-pulldown at CTS1-6 was 19.2%, corresponding to a 2.9-fold reduction compared to that of the untreated cells. Fluorophore detection using cell cytometry revealed that, 9 days after transfection, scFv-TET1CD expression was lost; hence, active epigenome editing must have stopped at this timepoint the latest (Supplementary Figure S2). For this reason, the data presented in this paper document a stable reprogramming of the chromatin state of ICR1.

### 3.2. Targeted Demethylation of the H19/IGF2 ICR1 Analyzed by Bisulfite Sequencing

**Figure 4:**
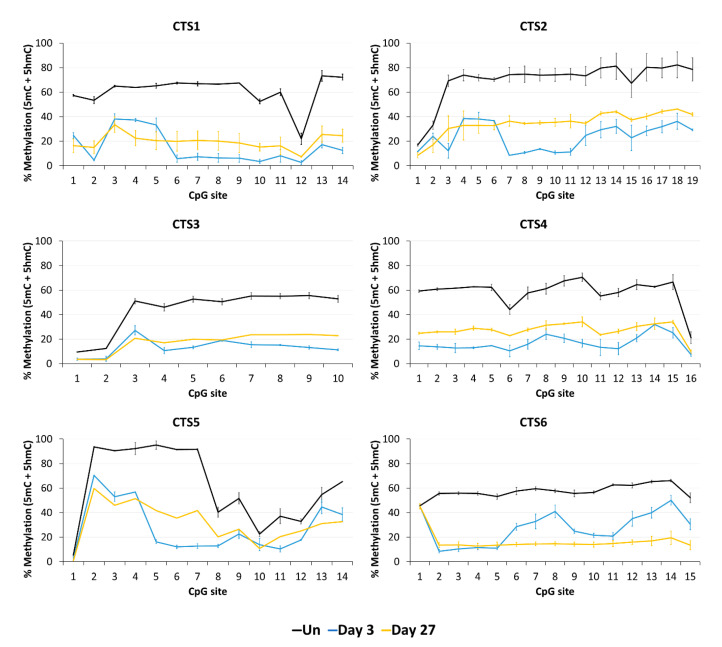
DNA methylation at the CTCF target site (CTS) regions determined by BS-seq. The level of DNA methylation, which in standard BS-seq includes 5mC plus 5hmC, is shown at each CpG site of the respective BS-PCR amplicons. The graphs show the DNA methylation of untreated HEK293 cells (Un, black line) and of cells on day 3 (blue line) and on day 27 (yellow line) after transfection with the EpiEditor. Data are represented as the mean of two independent biological replicates ± SD. For CTS3 and CTS5 on day 27, only one biological replicate is shown.

Next, we aimed to confirm the stable DNA demethylation up to 27 days and determine the precise methylation profiles at the target region with CpG site resolution by bisulfite sequencing (BS-seq). For this, HEK293 cells were transfected and sorted by FACS three days after transfection. On the same day (day 3), a part of the cells was harvested for gDNA isolation and the remaining cells were cultivated until day 27. The isolated DNA was bisulfite-converted and amplicons for NGS were generated from CTS1-6. The methylation levels at individual CpG sites within the sequencing amplicons are displayed in Figure 4.

**Figure 5:**
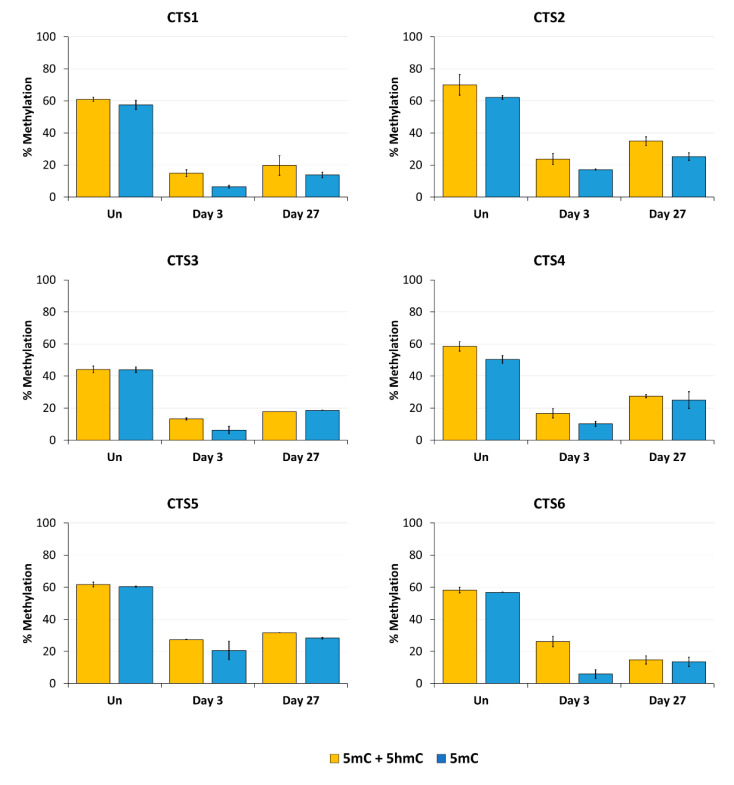
DNA modification analysis at the CTCF target site (CTS) regions determined by BS-seq and oxBS-seq. The average methylation over all CpG sites of the BS-PCR amplicons is shown in yellow (5mC + 5hmC). The 5mC levels in blue are also shown as the average methylation over all CpG sites of the oxBS-PCR amplicons. The 5mC + 5hmC data were taken from Figure 4. The 5mC data are shown as the mean of two independent biological replicates ± SD.

In agreement with the findings of the MBD2-pulldown assay, the average DNA methylation over all CpG sites within the sequenced amplicon was reduced at all CTSs compared to the untreated cells at day 3 after transfection (Figure 5). The weakest effect was seen at CTS6 with 55% demethylation and the strongest effect at CTS1 with 76% demethylation compared to the untreated cells. The relative methylation loss was maintained until day 27, when still 68% demethylation was observed at CTS1. At CTS6, the methylation level decreased even further and was 75% demethylated at day 27 compared to the untreated cells.

To investigate the locus specificity of the targeted DNA demethylation, the methylation levels at four untargeted regions have been analyzed as well: at the *H19* promoter, at a region in the *IGF2* gene, and at two randomly selected highly methylated genomic regions considered as off-target control regions, *FANCB* and *SLC6A3* (Supplementary Figure S3). The data showed that some off-target demethylation occurred at the *H19* promoter. An approximately 32% reduction in DNA methylation was observed and the effect remained stable until day 27. The *IGF2* region was initially unmethylated and any off-target demethylation observed was within experimental fluctuations. At the *FANCB* locus, no untargeted demethylation was observed, but at the *SLC6A3* locus, some weak DNA demethylation was detected (14% on day 3 after transfection and 9% on day 27).

### 3.3. Analysis of the Targeted Demethylation of the H19/IGF2 ICR1 by Oxidative Bisulfite Sequencing

DNA demethylation catalyzed by TET1 is initiated by the generation of 5hmC, the product of the first step of the iterative oxidation of 5mC. Like 5mC, 5hmC plays a role in the regulation of gene expression [33]. With standard BS-seq, 5mC and 5hmC cannot be discriminated [34]. Therefore, we applied oxidative bisulfite sequencing (oxBS-seq) to obtain information about the actual 5mC and 5hmC levels at our target regions. In oxBS, an oxidation step of 5hmC to 5fC precedes the bisulfite conversion, in which 5fC is then converted to uracil [35]. Therefore, by the comparison of the results of BS-seq (allowing to distinguish 5mC and 5hmC from unmethylated C) and oxBS-seq (allowing to distinguish 5mC from 5hmC and unmethylated C), the relative amounts of 5mC and 5hmC can be deduced.

Our data clearly illustrate (Figure 5) that most CTSs showed an increase in 5hmC levels compared to the untreated cells 3 days after transfection, which reflects the catalytic activity of TET1CD. This effect was reduced 27 days after transfection, which was expected because there is no established mechanism for the maintenance of 5hmC. The analysis of the corrected 5mC levels deduced from this experiment confirmed the previous findings as the overall 5hmC levels were relatively low. Three days after transfection, 5mC levels were reduced between 66% at CTS5 and 90% at CTS1 and CTS6 compared to the untreated cells. The strongest persistence of the demethylated state was seen at CTS1 and CTS6. After 27 days, these CTSs were still 76% demethylated compared to the untreated cells.

### 3.4. CTCF Occupancy Is Increased after DNA Demethylation at the H19/IGF2 ICR1

**Figure 6:**
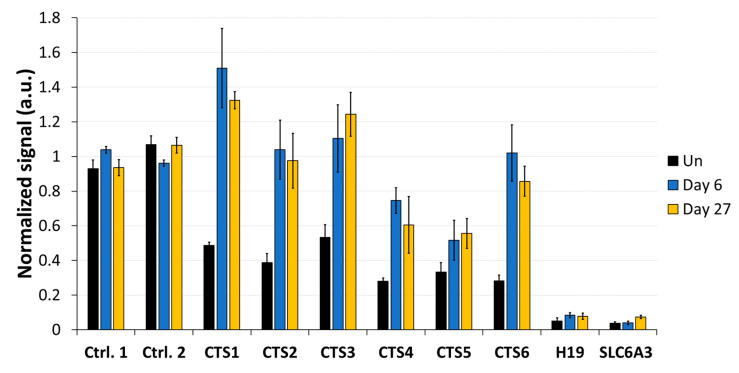
CTCF binding analysis at the CTCF target sites (CTS) of the *H19*/*IGF2* ICR1. CTCF-ChIP-qPCR signals were normalized to input DNA and to the average of two positive control regions. After transfecting HEK293 cells with the EpiEditor, the cells were harvested on day 6 and day 27. ChIP experiments were performed with an antibody against CTCF and an IgG control (Supplementary Figure S4). Analysis of CTCF levels was conducted at CTS1-6 and two negative control regions within the *H19* gene body and *SLC6A3* promoter. Un: untreated HEK293 cells. Data are shown as the mean of three independent biological replicates for “Un” and “Day 6” and of two independent biological replicates for “Day 27” ± SD. a.u. = arbitrary unit.

To determine whether DNA demethylation at CTSs affects the binding of CTCF, we performed ChIP with an αCTCF antibody and analyzed the pulldown by qPCR. HEK293 cells were transfected and sorted by FACS, as described above, and ChIP experiments were conducted on day 6 and on day 27 after transfection. ChIP-qPCR data were normalized to input, followed by an internal normalization to the average of two control CTCF target sites (positive control regions 1 and 2). These control sites were selected based on the strong CTCF enrichment found in publicly available data [36] as well as their low CpG density, suggesting that an eventual unspecific TET1 activity would not affect the outcome. In the untreated cells, the average normalized signal at CTS1-6 was 0.38 (Figure 6). After DNA demethylation with TET1CD, a strong increase in CTCF occupancy at the CTSs was observed on day 6, ranging between a 1.6-fold increase at CTS5 and a 3.6-fold increase at CTS6 compared to the untreated cells. The average normalized signal at CTS1-6 was 0.99 on day 6, corresponding to an average fold-change of 2.6 compared to the untreated cells. This increased CTCF occupancy remained stable until day 27 after transfection, when the average ChIP-qPCR signal at CTS1-6 was still 2.4-fold increased.

### 3.5. DNA Demethylation at the ICR1 Leads to Changes in mRNA Expression

**Figure 7:**
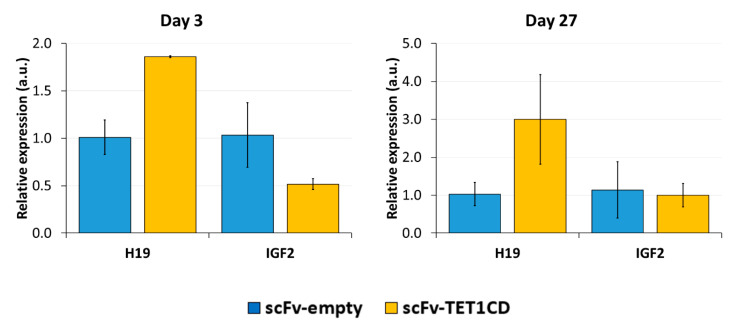
Expression of *H19* and *IGF2* after targeted DNA demethylation analyzed by RT-qPCR. Relative expression of *H19* and *IGF2* to the housekeeping gene *SDHA* and to the control treatment “scFv-empty” is shown on day 3 and day 27 after the transfection of the EpiEditor. The control transfection with dCas9-SunTag and sgRNAs, but no effector domain, was used as control to exclude that changes in expression were caused by the transfection procedure or the binding of dCas9-SunTag to the target region. Data are represented as the normalized −ΔΔCq ± SD of three independent biological replicates. For “Day 3, scFv-TET1CD”, only two biological replicates were performed. a.u. = arbitrary unit.

Lastly, we analyzed whether DNA demethylation, as well CTCF binding to the unmethylated CTSs, affected the expression of the *H19* and *IGF2* genes by RT-qPCR (Figure 7). Since half of the alleles are expected to express *H19* in the initial state, a complete demethylation could lead to a 2-fold increase in expression. On day 3 after transfection with dCas9-SunTag-scFv-TET1CD and the sgRNAs, a 1.8-fold increase in the relative *H19* expression was observed compared to the cells transfected with dCas9-SunTag-scFv-empty and the sgRNAs, which suggests an almost complete reprogramming of *H19* expression at this locus. This increase remained stable until day 27. Contrarily, the relative expression of *IGF2* was 2-fold reduced. However, after 27 days, the relative *IGF2* expression returned to a similar level to that of the control transfected cells, indicating that the silencing of *IGF2* was not stable.

## 4. Discussion

Imprinting is a paradigmatic epigenetic process in which allele-specific DNA methylation at ICRs established in the germ line regulates the allele-specific expression of a set of associated genes in the next generation. Abnormal imprinting can cause rare human diseases, which in principle could be tackled by the targeted rewriting of the DNA methylation at the ICR. Hence, the reprogramming of imprinted loci has interesting clinical applications, but it is also useful for fundamental research. In this study, we conducted a pilot study and successfully demethylated CTCF target sites within the *H19/IGF2* ICR1 in human HEK293 cells. We observed the specific generation of 5hmC at the target regions followed by an up to 90% reduction in the 5mC level achieved on day 3 after transfection. Although the expression of the scFv-TET1CD was lost 9 days after transfection, DNA demethylation was stable up to 27 days at the CTS1-6 (the last time point investigated by us), demonstrating the stable epigenome reprogramming of the locus. If one assumes that active editing stopped on day 9 (which is a conservative estimation, as all components must be present in order for the EpiEditor to be active), demethylation was stable for at least 18 days, corresponding to 14–18 cell divisions. Consistent with the robust demethylation, a stable increase in bound CTCF at CTS1-6 was shown and *H19* expression was increased until day 27. These findings indicate that not only the DNA methylation was reprogrammed, but the chromatin state of the ICR1 region was altered from a “paternal” (methylated) state in half of the alleles before treatment to a “maternal” (unmethylated) state with CTCF binding in all alleles after treatment. Related to this, it would be interesting to investigate in future if the ICRs change their position in the cell nucleus after epigenome editing, which could be detected by fluorescence in situ hybridization (FISH).

In contrast to the stable expression changes of *H19*, the expression of *IGF2* was reduced only transiently on day 3, but after 27 days, it returned to the expression level observed in the empty-vector control transfection. This effect may represent a secondary response of the cells to reprogramming during continuous culturing, where selective pressure may enrich cells that re-express the IGF2 growth factor and, therefore, propagate faster. In follow-up studies, it would be interesting to study transient and stable gene expression changes at global level after epigenome editing by RNA-seq.

In addition to a robust reprogramming mediated by the epigenome editing tool, it is important to minimize off-target activities, especially if the editors are to be used in clinical applications. We observed low off-target activities at distal sites, which were mostly transient compared to the robust on-target editing. Within the ICR, we observed strong demethylation effects at CTS4 and CTS5, which are targeted by sgRNA5 with two mismatches. Interestingly, we observed a partial DNA demethylation at the *H19* promoter at a distance of more than 1 kb away from the closest sgRNA-binding site, which was more pronounced than the off-target effects observed at the other two tested loci. This finding may be explained by the fact that the ICR1 and *H19* promoter are placed in one topologically associated domain (TAD) in the original ICR1-methylated state when no CTCF is bound [37]. Therefore, the catalytic domains of TET1 targeted to the ICR1 may also have access to the *H19* promoter due to the flexibility of the targeting device and the chromatin.

Our data are in general agreement with the basic findings of previous studies [15,25], but they also provide additional details regarding the reprogramming of the *H19*/*IGF2* ICR1 in human HEK293 cells, including the analysis of the CpG site-specific profiles of 5mC and 5hmC and an analysis period of up to 27 days, which provide important new mechanistic insights.

## Data Availability

BS-seq and oxidative BS-seq data as well as the sequence of the dCas9-5xSunTag-scFv-TET1CD plasmid are available at https://doi.org/10.18419/darus-3790.

## Supplementary Materials

The following supporting information can be downloaded at: https://www.mdpi.com/article/10.3390/genes15010080/s1.
