# Supplementary material for: Locus-Specific and Stable DNA Demethylation at the H19/IGF2 ICR1 by Epigenome Editing Using a dCas9-SunTag System and the Catalytic Domain of TET1
Source: Genes (Basel). 2024 Jan 8;15(1):80. doi: 10.3390/genes15010080 (PMC10815749; doi:10.3390/genes15010080)
Supplement: Supplementary file 1 [file genes-15-00080-s001.zip › genes-2807012-supplementary.pdf]

Locus-specific and stable DNA demethylation at the H19/IGF2 ICR1 by epigenome editing using a dCas9-Suntag system and the catalytic domain of TET1

Claudia Albrecht, Nivethika Rajaram, Julian Broche, Pavel Bashtrykov, and Albert Jeltsch*

Supplementary Information

Supplementary figures and tables

Supplementary Figure S1. Scheme of the used EpiEditor plasmids

Supplementary Figure S2. Median GFP fluorescence signals measured by FACS at different time points after transfection

Supplementary Figure S3. DNA methylation at the *H19*, *IGF2*, *FANCB* and *SLC6A3* promoters determined by BS-seq

Supplementary Figure S4. Control CTCF ChIP-qPCR signals normalized to input DNA and to the average of two positive control regions

Supplementary Table S1: SgRNA sequences and primer pairs used in this study for MBD2-pulldown-qPCR, CTCF-ChIP-qPCR and RT-qPCR

Supplementary Table S2: Primer pairs used for BS-seq and genomic positions of the BS-seq amplicons

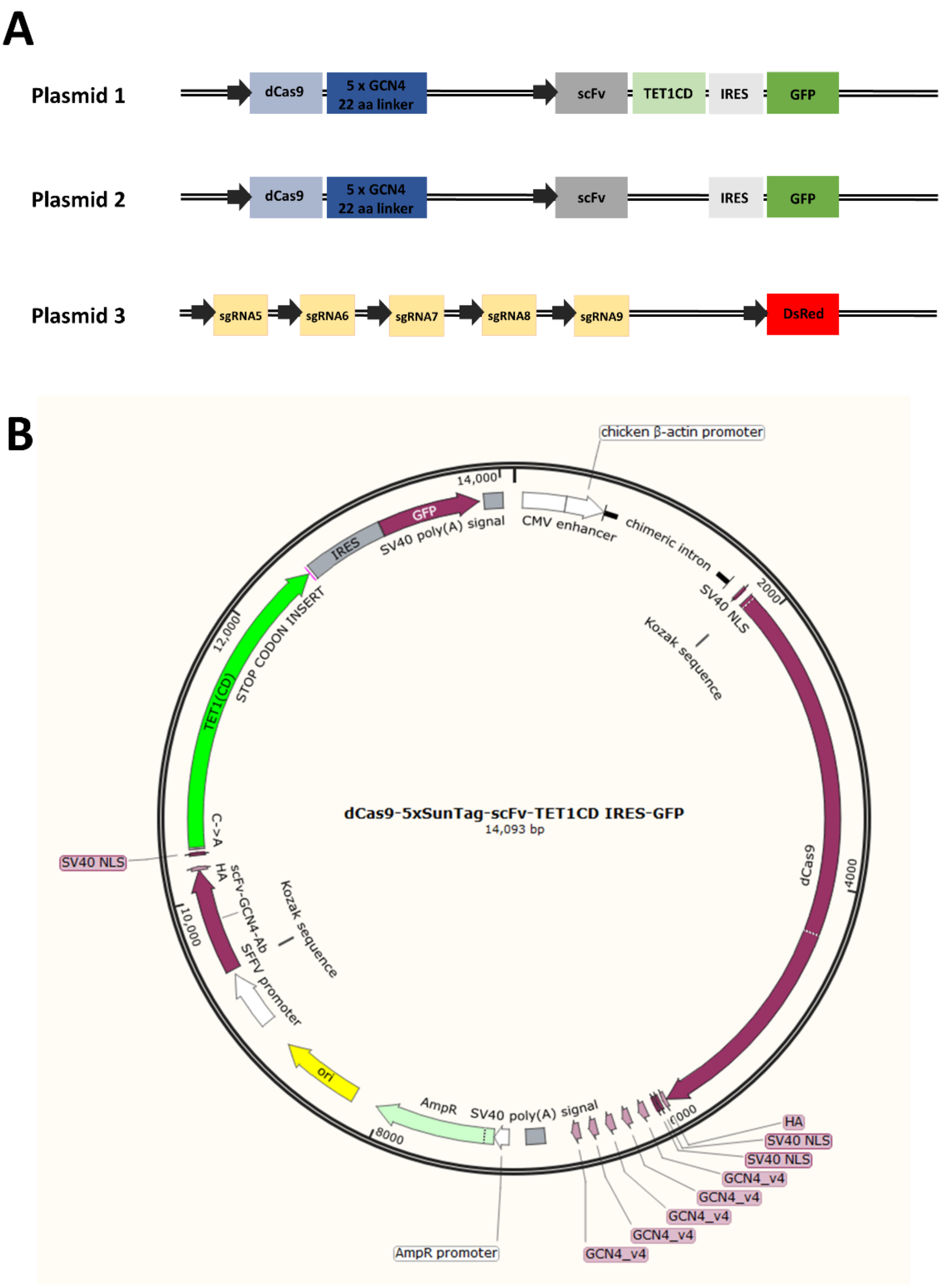

Supplementary Figure S1. Scheme of the used EpiEditor plasmids. (**A**) Scheme of the functional parts of the three plasmids used for transfection into HEK293 cells. The first plasmid (Plasmid 1) includes genes for dCas9 coupled to the SunTag consisting of five GCN4 repeats separated by 22 amino acid (aa) long linkers. The co-expression of scFv-TET1CD-IRES-GFP is controlled by a separate promoter. The translation of GFP is initiated by an internal ribosome entry site (IRES). The second plasmid (Plasmid 2) is similar to Plasmid 1, but does not contain an effector domain (scFV-empty). Plasmid 2 was transfected into HEK293 cells as a negative control treatment. Plasmid 3 contains five different sgRNAs (sgRNA5-sgRNA9). Every sgRNA is flanked by an U6 promoter and a gRNA scaffold. In addition, the DsRed fluorophore is expressed under a CMV promoter. (**B**) Map of Plasmid 1 (created with SnapGene software, www.snapgene.com). The sequence of this plasmid is available at https://doi.org/10.18419/darus-3790.

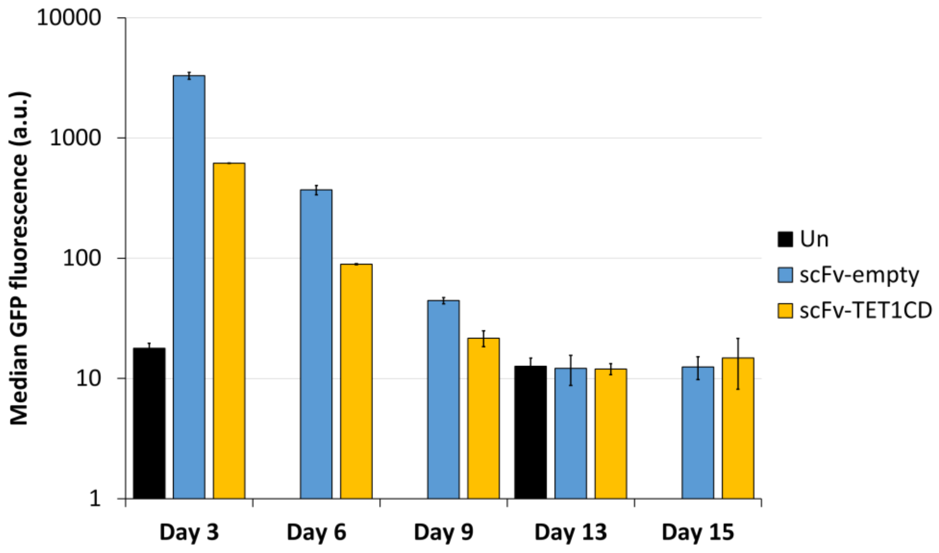

Supplementary Figure S2. Median GFP fluorescence signals of HEK293 cells measured by FACS at different time points after transfection. HEK293 cells were transiently co-transfected with the two plasmids required for epigenome editing. On day 3 after transfection, cells showing both GFP and DsRed fluorescence were sorted using a cell sorter. Using FACS, the GFP fluorescence was measured in untreated cells (Un) as well as in a part of the transfected and sorted cells (scFv-TET1CD). In addition, the GFP intensity was measured in sorted cells transfected with the control plasmid lacking an effector domain (scFv-empty). The remaining cells were cultivated and GFP intensities were measured again on day 6, day 9 and day 15 after transfection. Data are represented as the mean of three independent biological replicates for Un and scFv-empty, as well as two independent biological replicates for scFv-TET1CD ± SD. a.u. = arbitrary unit.

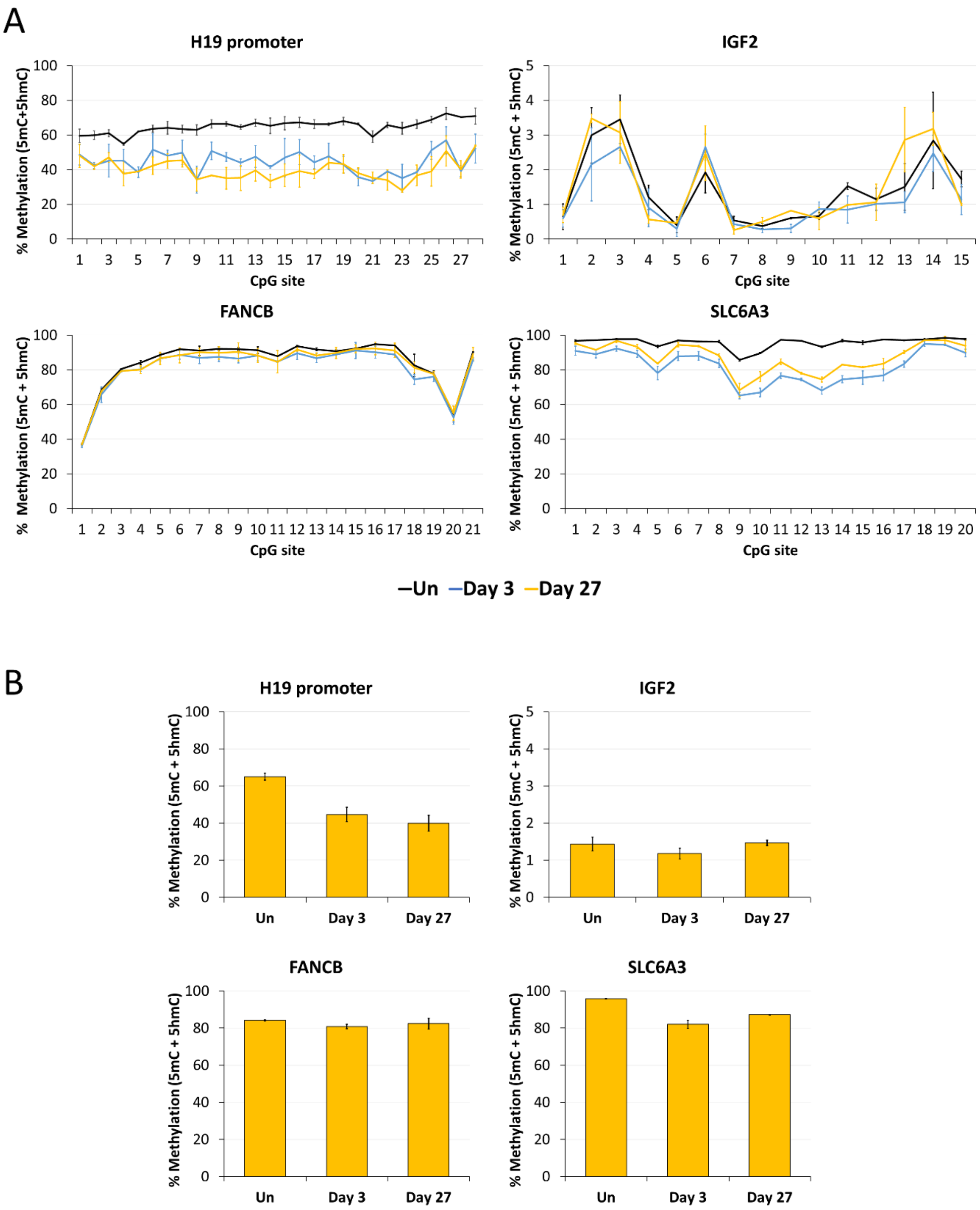

Supplementary Figure S3. DNA methylation at the *H19*, *IGF2*, *FANCB* and *SLC6A3* promoters determined by BS-seq. (**A**) Percentage of methylation, which includes 5mC plus 5hmC levels, is shown at each CpG site of the respective BS-PCR amplicons. Shown are DNA methylation levels of untreated HEK293 cells (Un, black line) and of cells on day 3 (blue line) and on day 27 (yellow line) after transfection with the EpiEditor. Data are represented as mean of two independent biological replicates ± SD, except for H19 promoter "Un", IGF2 "Day 3", FANCB "Day 3" and SLC6A3 "Day 3", which are represented as mean of three independent biological replicates ± SD. (**B**) Average percentage of DNA methylation over all CpG sites shown in panel (A).

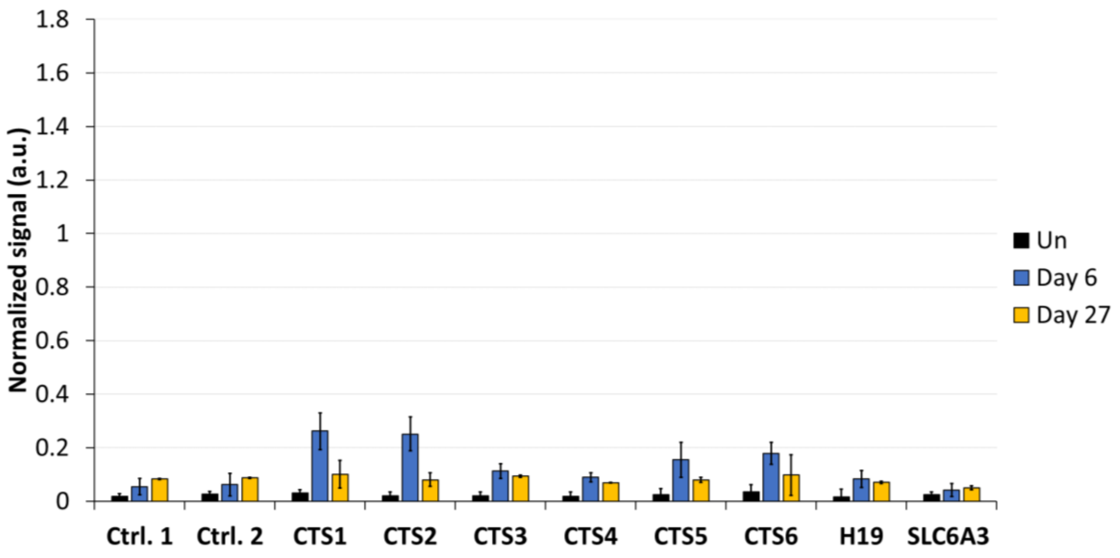

Supplementary Figure S4. Control CTCF ChIP-qPCR signals normalized to input DNA and to the average of two positive control regions. After transfecting HEK293 cells with the EpiEditor, cells were harvested on day 6 and day 27. Control-ChIP experiments were performed simultaneously to the CTCF-ChIP experiments using an IgG control antibody. CTCF-levels were analyzed at CTS1-6, at the *H19* gene body and the *SLC6A3* promoter. Un: untreated HEK293 cells. Data are shown as mean of 3 independent biological replicates for "Un" and "Day 6" and of 2 independent biological replicates for "Day 27" ± SD. a.u. = arbitrary unit.

Supplementary Table S1: SgRNA sequences and primer pairs used in this study for MBD2-pulldown-qPCR, CTCF-ChIP-qPCR and RT-qPCR.

sgRNA sequences	
**sgRNA5**	CCCCAGTTTGGGCGGGCTC
**sgRNA6**	TCTCACCGCCTGGATGGCA
**sgRNA7**	GGGCGAACCCCATCCAGGG
**sgRNA8**	CGCCCCGATGGTGCAGAAT
**sgRNA9**	TGCCCTGATGGCGCAGAAT

Primers for qPCR after MBD2-PD and CTCF-ChIP		
**CTS1**	FP	TGGCGCAGAATCGGCTGTAC
	RP	GAGACCTGGGACGTTTCTGT
**CTS2**	FP	GCCCCGATGGTGCAGAATC
	RP	CGGCACCTAGCTTGCGT
**CTS3**	FP	GATGGCACAGAATCGGTTGTAAG
	RP	CATCCAGGGAGGGCTTGG
**CTS4**	FP	ACCCGGATGGTGCAGAATTG
	RP	CGCCTGGCTTGCGGGA
**CTS5**	FP	GGTTGTAGTTGTGGAATCAGAGG
	RP	CCCGGCTTGGATGACCT
**CTS6**	FP	ACCGCCTGGATGGCACG
	RP	TGAACCCTGCGACGCGT
**H19 gene body**	FP	GTCATGTCCTGCTTGTCACG
	RP	TTCTCCCCACACGACTCTCT
**SLC6A3**	FP	GCACTCGCCTAAGAAAACCA
	RP	GGAAGGAAAGCCTCGGAGT
**pos. control 1**	FP	CTCATTCTCAGCCCTCACGC
	RP	CCTGTTTTTCTTTGAAATCGTCCAC
**pos. control 2**	FP	TGCCGAGAACGTGTGACTC
	RP	CTGCGGTATTTGCAGCAGTA

Primers for RT-qPCR		
**H19**	FP	GATGGTGTCTTTGATGTTGGGCTGA
	RP	CTTTACAACCACTGCACTACCTGAC
**IGF2**	FP	TCCAGGTGTCATATTGGAAGAACT
	RP	CAAGTCCGAGAGGGACGTG
**SDHA**	FP	TGGGAACAAGAGGGCATCTG
	RP	CCACCACTGCATCAAATTCAT

Supplementary Table S2: Primer pairs used for BS-seq and genomic positions of the BS-seq amplicons. NNNNN represents a unique molecular identifier sequence comprising five randomized bases, which are needed for the sequencing reaction.

PCR1 primers for BS/oxBS-seq			Tm [°C]	Genomic position (GRCh38/hg38)
**CTS1**	FP	ACACTCTTTCCCTACACGACGCTCTTCCGATCTNNNNNATATCAGGTATTTTTGGAGGTTTTTTATTTAG	57	chr11: 2,002,904-2,003,171
	RP	GTGACTGGAGTTCAGACGTGTGCTCTTCCGATCTNNNNNGAGACTATCTATCTCTAACAACCCTC		
**CTS2**	FP	ACACTCTTTCCCTACACGACGCTCTTCCGATCTNNNNNTGTGAGAGGTGTTTTAGTTTTTTGGATGATA	56	chr11: 2,002,488-2,002,811
	RP	GTGACTGGAGTTCAGACGTGTGCTCTTCCGATCTNNNNNCGCAGACTCCCATAAATATTCTATCCCTCACTA		
**CTS3**	FP	ACACTCTTTCCCTACACGACGCTCTTCCGATCTNNNNNATCGCGGGGAGATGAGATATTTTGGTGATAATG	50	chr11: 2,002,171-2,002,437
	RP	GTGACTGGAGTTCAGACGTGTGCTCTTCCGATCTNNNNNGACGACCCCATCCAAAAAAAACTTAAAC		
**CTS4**	FP	ACACTCTTTCCCTACACGACGCTCTTCCGATCTNNNNNTATGTTATAGGGTTTTTGGTAGGTTTA	56	chr11: 2,000,656-2,000,922
	RP	GTGACTGGAGTTCAGACGTGTGCTCTTCCGATCTNNNNNCGCGCCCCATAAATATCCTATCCCTAATA		
**CTS5**	FP	ACACTCTTTCCCTACACGACGCTCTTCCGATCTNNNNNATCAGTTTAGGGTGAGATTTTTTTTG	56	chr11: 2,000,247-2,000,534
	RP	GTGACTGGAGTTCAGACGTGTGCTCTTCCGATCTNNNNNGTGAGACTCCCATAAATATCCTATACCTC		
**CTS6**	FP	ACACTCTTTCCCTACACGACGCTCTTCCGATCTNNNNNTGTATGGTAYGGAATTGGTTGTAGTTGTGG	59	chr11: 1,999,850-2,000,018
	RP	GTGACTGGAGTTCAGACGTGTGCTCTTCCGATCTNNNNNCTAGCATATCCTATTCCCAAATAACCCC		
**H19 promoter**	FP	ACACTCTTTCCCTACACGACGCTCTTCCGATCTNNNNNTCGACTTGAGGGGTAGAGGGAAGTGT	50	chr11: 1,998,297-1,998,657
	RP	GTGACTGGAGTTCAGACGTGTGCTCTTCCGATCTNNNNNCTACTCTCACCAAAAACCAAAATAATAAC		
**IGF2**	FP	ACACTCTTTCCCTACACGACGCTCTTCCGATCTNNNNNAGCATGGTAGAGATAGTGGGAGAGATAGAGTGAA	56	chr11: 2,138,527-2,138,739
	RP	GTGACTGGAGTTCAGACGTGTGCTCTTCCGATCTNNNNNCAGATCCCCCCCAACTCCTAACCTC		
**SLC6A3**	FP	ACACTCTTTCCCTACACGACGCTCTTCCGATCTNNNNNCTGACGAGGTTTTTTAGGTTTAGTTTT	50	chr5: 1,446,051-1,446,276
	RP	GTGACTGGAGTTCAGACGTGTGCTCTTCCGATCTNNNNNTACGCCCTAAAAACTCCATTCCTCC		
**FANCB**	FP	ACACTCTTTCCCTACACGACGCTCTTCCGATCTNNNNNTACGAGGYGGAGTTTAGAAGTTAGTTAGG	50	chrX: 14,873,104-14,873,323
	RP	GTGACTGGAGTTCAGACGTGTGCTCTTCCGATCTTGAACCCTCAACCTAAATCCCATT		
